# Factors Associated with Adenoma Detection Rate and Diagnosis of Polyps and Colorectal Cancer during Colonoscopy in France: Results of a Prospective, Nationwide Survey

**DOI:** 10.1371/journal.pone.0068947

**Published:** 2013-07-18

**Authors:** Maximilien Barret, Christian Boustiere, Jean-Marc Canard, Jean-Pierre Arpurt, David Bernardini, Philippe Bulois, Stanislas Chaussade, Denis Heresbach, Isabelle Joly, Jean Lapuelle, René Laugier, Gilles Lesur, Patrice Pienkowski, Thierry Ponchon, Bertrand Pujol, Bruno Richard-Molard, Michel Robaszkiewicz, Rémi Systchenko, Fatima Abbas, Anne-Marie Schott-Pethelaz, Christophe Cellier, for the Société Française d'Endoscopie Digestive

**Affiliations:** 1 Department of Gastroenterology and Digestive Endoscopy, George Pompidou European Hospital and Faculté Paris Descartes, Paris, France; 2 Department of Gastroenterology, Saint Joseph Hospital, Marseille, France; 3 Department of Gastroenterology, Henri Duffaut Hospital, Avignon, France; 4 Department of Gastroenterology, Toulon Font Pré Hospital, Toulon, France; 5 Department of Gastroenterology, Huriez Hospital, Lille, France; 6 Department of Gastroenterology, Cochin Hospital, Paris, France; 7 Department of Gastroenterology, Cannes Hospital, Cannes, France; 8 Department of Gastroenterology, Saint-Brieuc Private Hospital, Saint Brieuc, France; 9 Department of Gastroenterology, Saint Jean Languedoc Private Hospital, Toulouse, France; 10 Department of Gastroenterology, La Timone Hospital, Marseille, France; 11 Department of Gastroenterology, Ambroise Paré Hospital, Boulogne, France; 12 Department of Gastroenterology, Le Pont de Chaume Private Hospital, Montauban, France; 13 Department of Gastroenterology, Edouard Herriot Hospital, Lyon, France; 14 Department of Gastroenterology, Jean Mermoz Private Hospital, Lyon, France; 15 Department of Gastroenterology, Saint Martin Private Hospital, Pessac, France; 16 Department of Gastroenterology, La Cavale Blanche University Hospital, Brest, France; 17 Gastroenterologist, Irigny, France; 18 Pôle information médicale évaluation recherche, Université Claude Bernard-Lyon 1, Lyon, France; National Cancer Center, Japan

## Abstract

**Introduction:**

Colonoscopy can prevent deaths due to colorectal cancer (CRC) through early diagnosis or resection of colonic adenomas. We conducted a prospective, nationwide study on colonoscopy practice in France.

**Methods:**

An online questionnaire was administered to 2,600 French gastroenterologists. Data from all consecutive colonoscopies performed during one week were collected. A statistical extrapolation of the results to a whole year was performed, and factors potentially associated with the adenoma detection rate (ADR) or the diagnosis of polyps or cancer were assessed.

**Results:**

A total of 342 gastroenterologists, representative of the overall population of French gastroenterologists, provided data on 3,266 colonoscopies, corresponding to 1,200,529 (95% CI: 1,125,936-1,275,122) procedures for the year 2011. The indication for colonoscopy was CRC screening and digestive symptoms in 49.6% and 38.9% of cases, respectively. Polypectomy was performed in 35.5% of cases. The ADR and prevalence of CRC were 17.7% and 2.9%, respectively. The main factors associated with a high ADR were male gender (p=0.0001), age over 50 (p=0.0001), personal or family history of CRC or colorectal polyps (p<0.0001 and p<0.0001, respectively), and positive fecal occult blood test (p=0.0005). The prevalence of CRC was three times higher in patients with their first colonoscopy (4.2% *vs*. 1.4%; p<0.0001).

**Conclusions:**

For the first time in France, we report nationwide prospective data on colonoscopy practice, including histological results. We found an average ADR of 17.7%, and observed reduced CRC incidence in patients with previous colonoscopy.

## Introduction

Colorectal cancer (CRC) is the third most common cancer in both men and women worldwide, with 40,500 new cases diagnosed in France in 2011 [1]. Currently in France, patients at average risk for CRC (above 50 years of age and without personal or familial history of CRC or colorectal adenomas) are screened with guaiac-based fecal occult blood test (FOBT) repeated every two year, and subsequent colonoscopy only in case of positive FOBT [2]. Among the various diagnostic tools available, colonoscopy enables clinicians to simultaneously screen and prevent CRC by removing adenomatous polyps, as recently confirmed by the lower mortality from CRC observed in patients with previous polypectomy [3]. Nevertheless, the number and age of screened patients are increasing. Less costly and/or less invasive CRC screening tools, such as CT colonography [4] and flexible sigmoidoscopy [5] are available. Therefore, the diagnostic yield of colonoscopy needs to be precisely assessed and optimized. In this field, the vast majority of reported data come from North America, and data from Europe are relatively scarce [6–10]. However, CRC features can differ between populations [11], and CRC screening should rely as much as possible on local data. Here, a nationwide, prospective survey of colonoscopies performed by French gastroenterologists was conducted to assess the diagnostic yield of this procedure in 2011. We focused on the adenoma detection rate (ADR) and sought to identify factors associated with changes in ADR.

## Methods

An online questionnaire was sent to 2,600 gastroenterologists who are members of the French Society of Digestive Endoscopy. Demographic and professional information was collected from each participant, including age, gender, type of practice (private or public), type of healthcare facility (university hospital, community hospital, private hospital), and geographic area of activity. These data were compared to those of the French College of Physicians for the year 2011. Data from all consecutive gastrointestinal endoscopies performed during a week were recorded online. The week was randomly selected outside school holidays. For each colonoscopy, the anesthetic modalities, the American Society of Anesthesiology (ASA) score and the intake of antiplatelet or anticoagulant drugs were recorded, as well as data on the procedure itself (type and efficacy of bowel preparation, indication, cecal intubation rates, findings and therapeutic interventions, histological analysis of the resected polyps, and complications). The diagnostic yield of colonoscopy was assessed through the polyp detection rate, the ADR, and the CRC detection rate. The ADR was defined as the number of colonoscopies revealing at least one adenomatous lesion divided by the total number of colonoscopies performed [12].

Statistical analysis and extrapolations were performed with SAS V 9.2 (SAS Institute Campus Drive, Cary, NC, USA) and STATA V 11.0 (Stata Corp, College Station, Texas, USA), respectively. Categorical data were displayed using proportions and compared by Chi-square test or Fisher’s exact test as appropriate. Continuous data were displayed using means ± standard deviation (SD) or medians (range) according to their distribution. The survey population of 342 gastroenterologists was considered a sample of the overall population of French gastroenterologists who reported a regular activity in digestive endoscopy for the year 2011, corresponding to 2,600 gastroenterologists. Binomial tests were performed to compare the main characteristics of our sample to 2011 data from the French College of Physicians regarding gastroenterologists. We hypothesized that the total number of patients included reflected the number of endoscopies performed in a week by the participating gastroenterologists. The extrapolated number of colonoscopies is the product from the number of colonoscopies during one week of the survey stratified by the type of gastroenterology practice (public hospitals and private practitioners), with the weight of gastroenterologists relative to the overall population of 2,600 gastroenterologists for the year 2011 in each stratum [13]. The final extrapolated number of colonoscopies was multiplied by the number of working weeks for each stratum of gastroenterologists, corresponding to 49 weeks for the public hospital gastroenterologists and 48.2 weeks for private practitioners. For each main characteristic ADRs were compared by using a chi-square test. To test the association and the trend of the ADR with quantitative variables, a likelihood-ratio test was performed. Multivariate analysis of the ADR was preformed using a logistic regression. A p value of less than 0.05 was considered statistically significant for all comparisons.

### Ethics statement

The study was designed by the French Society of Digestive Endoscopy (Société Française d’Endoscopie Digestive, SFED). The SFED institutional review board (“Commission Juridique de la SFED”) specifically approved this study. Since the study was strictly observational and used anonymous data, the institutional review board from the SFED, in accordance to the laws that regulate “non interventional clinical research” in France, namely articles L.1121-1 and R.1121-2 of the Public Health Code, did not require the written informed consent from the participants or the authorization from any other ethics committee to conduct this survey.

## Results

### Participating physicians and patients

Between November 28 and December 4, 2011, a total of 475 gastroenterologists completed the survey, and 342 (13.2%) provided analyzable data on 5,069 patients. Colonoscopies were performed on 3,266 patients, corresponding after extrapolation to a total of 1,200,529 colonoscopies (95% confidence interval (CI): 1,125,936-1,275,122) for the year 2011. In terms of gastroenterologist age (age <40 years: 12.6% vs. 14.9%; age ≥ 55 years = 35.4% vs. 35.3% respectively, p (global) = 0.44), gender (male sex: 74.9% vs. 73.4%, respectively, p = 0.46), and geographic area of activity (p = 0.14), no statistically significant difference was noted between our sample of gastroenterologists and data from the French College of Physicians for 2011; however, the proportion of private practitioners was higher in our sample (71.1% *vs.* 59.1%; p <0.0001).

Overall, 1572 (48.1%) patients were men and 2504 (76.7%) were older than 50. The main indications for colonoscopy were asymptomatic CRC screening (personal or family (in first degree relatives) history of colorectal polyp or cancer, positive fecal occult blood test (FOBT), and average-risk screening colonoscopy) in 1619 (49.6%) patients, abdominal symptoms (either change in bowel habit, constipation, diarrhea, or abdominal pain) in 775 (23.7%) patients, rectal bleeding in 496 (15.2%) patients, and inflammatory bowel disease (at initial work-up or follow-up) in 86 (2.6%) patients. Patients’ characteristics are presented in table 1.

**Table 1:** Patient characteristics (n=3,266).

**Male gender**	1,572 (48.1%)
**Mean age ± SD (years)**	58.9 ± 15.6
ASA* score ≤ 2	2,998 (91.8%)
**Antiplatelet or anticoagulant treatment**	366 (11.2%)
**Previous colonoscopy**	1,524 (46.7%)
**Indication for colonoscopy**	
CRC^+^ screening	1,619 (49.6%)
Personal history of polyp or CRC	494 (15.1%)
Familial history in first degree relatives of colorectal polyp or CRC	917 (28.1%)
Positive FOBT^≠^	146 (4.5%)
Abdominal symptoms	775 (23.7%)
Rectal bleeding	496 (15.2%)
Inflammatory bowel disease	86 (2.6%)

* American Society of Anesthesiologists

+ colorectal cancer

≠ fecal occult blood test

### Colonoscopy findings

A total of 2966 (90.8%) colonoscopies were performed under general anesthesia. These were conducted in 1711 (52.4%) cases by an anesthesiologist, in 1235 (37.8%) cases by a trained nurse supervised by an anesthesiologist, and in 196 (0.6%) cases by the gastroenterologist him/herself. The ASA score was ≤ 2 for more than 90% of patients (2998/3266). A total of 366 (11.2%) patients were treated with antiplatelet or anticoagulant drugs, including aspirin in 52.5% (192/366) of cases and clopidogrel in 12.6% (46/366) of cases. Advice from a cardiologist was sought in one-third of cases (116/366), and antiplatelet therapy was modified in 53.1% (127/239) of patients. The bowel preparation consisted of either 4L of polyethylene glycol (PEG) alone or 2L of PEG with sodium ascorbate and additional fluid intake, sodium picosulfate or sodium phosphate (as tablet or solution) in 1579 (48.4%), 103 (3.2%), 702 (21.5%), and 467 (14.3%) cases, respectively. The other patients received mixed bowel preparations, and enemas were used in 225 (6.9%) patients. Split-dose regimens were used in 85.9% of cases, and the mean time elapsed between the last fluid intake and the colonoscopy was seven hours. Bowel cleansing was assessed with a specific score in 18.4% (601/3266) of cases. The bowel preparation was judged “unsatisfactory” in 7.9% (258/3266) of colonoscopies.

In 10.3% (336/3266) of cases, the colonoscopy did not reach the cecum. Forty-three percent (1404/3266) of colonoscopies were considered as strictly normal, and in 35.5% (1159/3266) cases at least one polyp was resected, either by polypectomy (30.9%, 1009/3266) or endoscopic mucosal resection (4.6%, 150/3266). Polyps were ≥ 10 mm in 7.4% (243/3266) of cases, numbered more than three in 5% (162/3266) of cases, and were located in the proximal colon (above the left colonic flexure) in 16.7% (545/3266) of cases. CRC diagnosis was established in 2.9% (94/3266) of cases, corresponding to a number of 34,624 (95%CI: 27,952-41,295) CRC for 2011 after extrapolation.

Histological analysis was available for 855 procedures (70.7% of the 1209 colonoscopies with histological material). The ADR was 17.7% (578/3266), and serrated adenomas were found in 1.4% (46/3266) of cases. A degenerated polyp with infiltrating adenocarcinoma was found in 0.4% (13/3266) of all colonoscopies. The ADR was 22.2% in the CRC screening patient group, 21.3% in the subgroup of patients with personal or familial (in first degree relatives) history of colorectal polyp or cancer, and 31.5% in patients with positive FOBT. The histological findings are presented in Table 2. The percentage of adenomas with high-grade dysplasia or carcinoma *in situ* and invasive carcinomas increased with the size of the largest polyp, from 1.7% (7/420) in lesions of 5 mm or less to 38.6% (17/44) in lesions larger than 20 mm, with a likelihood ratio of 45.96 (p<0.0001). The correlations between the size of the largest colonic polyp and the histological findings are presented in Table 3.

**Table 2:** Histological analysis of resected polyps.

Histology of resected polyps*	n - %**
Infiltrating adenocarcinoma	13 - 0.4%
**High-grade dysplasia in adenomas or carcinomas *insitu***	**59 - 1.8%**
**Benign or low-grade dysplasia in adenomas**	**47 - 14.5%**
**Serrated adenoma**	**46 - 1.4%**
Hyperplastic polyp	264 - 8.1%
Polyp without available histology	353 - 10.8%

* Only the most advanced polyp was taken into account

** Of the total number of colonoscopies performed (n=3266)

**Table 3:** Correlation between histology and size of largest polyp (n= 855).

**Histology**	**Size of largest polyp (S)**				p-value
	S ≤ 5 mm	6mm ≤ S ≤ 9 mm	10 mm ≤ S ≤ 20 mm	S >20 mm	
Hyperplastic, serrated, benign or low-grade dysplasia in adenomas	98.3% (413/420)	93.9% (231/246)	76.0% (92/121)	61.4% (27/44)	p<0.0001
High-grade dysplasia in adenomas, carcinoma *in situ*, infiltrating adenocarcinomas	1.7% (7/420)	6.1% (15/246)	24.0% (29/121)	38.6% (17/44)	

### Diagnostic yield of colonoscopy

Overall, the polyp detection rate was 35.5% (1159/3266). It was significantly higher in patients with a past history of colonoscopy (41.3% *vs.* 30.7%; p<0.001), and the previous colonoscopy was abnormal in 73.4% (352/479) of cases.

The ADR was 17.7% in our study. On univariate analysis, male gender (21.2% *vs.* 14.5%; p<0.0001), age over 50, (20.6% *vs.* 8.5%; p<0.0001), personal history of CRC or colorectal polyp (23.8% *vs.* 15.1%; p<0.0001), family history of CRC or colorectal polyp (21.7% *vs.* 16.2%; p=0.0002), and positive FOBT (28% *vs.* 17%; p=0.0008) were associated with higher ADR values. The ADR ranged from 20.1% or 20.2% with bowel preparations with sodium phosphate or PEG (2L or 4L protocols), respectively, to 14.8% with sodium picosulfate-based bowel preparations. The difference was statistically significant for both comparisons (p=0.002 for PEG *vs.* sodium picosulfate and p=0.02 for sodium phosphate *vs.* sodium picosulfate). The time of onset of the colonoscopy did not significantly affect the ADR. Among endoscopist-related characteristics, the age of the gastroenterologist did not affect the ADR (15.7% for <35 years old, 19.5% for 35 to 49 years old, and 17% for 50 years old and above, p= 0.18), but male practitioners had higher ADR than female practitioners (18.8% vs 12.7%, p=0.0005, respectively). On multivariate analysis, including patient sex and age, familial and personal history of colorectal polyps and CRC, positive FOBT, type of bowel preparation, and gender of endoscopist were all associated with statistically significant changes in the ADR, as shown on table 4.

**Table 4:** Multivariate analysis of factors associated with adenoma detection rate.

**Parameter**	**Odds Ratio (95% CI)**	**p**
**Male gender** (patient)	1.54 (1.27 – 1.85)	<.0001
**Age > 50 years**	2.35 (1.77 – 3.11)	<.0001
**Familial history (first degree relatives) of colorectal polyps or cancer**	1.48 (1.21 – 1.81)	0.0001
**Personal history of colorectal polyps or cancer**	1.92 (1.54 – 2.38)	<.0001
**Positive fecal occult blood test**	1.99 (1.35 – 2.93)	0.0005
**Type of bowel preparation**		
PEG* 2L (Moviprep®)	0.97 (0.54 – 1.77)	0.93
PEG* 4L (Colopeg®, Fortrans®, Klean-Prep®)	0.90 (0.62 – 1.32)	0.59
Sodium phosphate in tablets (Colokit®)	0.95 (0.59 – 1.53)	0.82
Sodium phosphate solution (Fleet-Phospho-Soda®)	0.75 (0.45 – 1.23)	0.25
Sodium picosulfate (CitraFleet®, Picoprep®)	0.64 (0.42 – 0.98)	0.04
**Male gender** (physician)	1.35 (1.03 – 1.78)	0.03

* polyethylene glycol

The prevalence of CRC was 4.2% (73/1742) in patients with no previous colonoscopy and 1.4% (21/1524) in patients with a past history of colonoscopy (p<0.0001). The previous colonoscopy was abnormal in more than 71.4% (1088/1524) of cases. In 14 cases, the colonoscopy had been performed within the past 60 months, suggesting an interval CRC rate of 0.4% (14/3266).

### Complications

Considering the 427,865 (95%CI: 389,108-466,622) polypectomies that were performed in France in 2011, the overall complication rate would be 1.1%. Hemorrhage, fever and intestinal perforation were reported in 0.6% (7/1159), 0.2% (2/1159) and 0.1% (1/1159) of cases, respectively. Patients’ age and gender, the intake of antiplatelet or anticoagulant drugs, a sessile/flat/pedunculated polyp type, and the localization of the polyps did not significantly affect the rate of complications. However, the rate of complications increased with the size of the resected polyps, from 0.2% (1/556) for polyps ≤ 5 mm to 8.2% (6/73) for polyps > 20 mm (likelihood ratio of 8.8; p=0.003). The only intestinal perforation reported in the present study was associated with a polypectomy.

## Discussion

This prospective, nationwide survey on colonoscopy practice, which included more than 3,000 procedures and histological findings, is the only one available in France to date. Thanks to the large representative sample of gastrointestinal endoscopists, the results could be extrapolated to a whole year. Our numbers are in accordance with those of the French National Health Insurance Fund, which reported that 1.28 million colonoscopies were actually performed in France in 2011; these were performed by private practitioners in 70% of cases, with 60% (vs. 56.7% in our survey) of patients aged 50 to 74, 5.5% (vs. 4.6% in our survey) of patients with positive FOBT, and 31.9% (vs. 35.5% in our survey) of patients with polyp resections. These data provide a retrospective confirmation that our gastroenterologist and colonoscopy sample was valid for extrapolation to the whole year of 2011 in France. However, histological data and adenoma detection rates are missing from this survey [14].

Among the 1,200,529 colonoscopies (95%CI: 1,125,936-1,275,122) that would have been performed in France during the year 2011, over one-third enabled the identification and resection of colorectal polyps, and 17.7% revealed at least one adenomatous lesion. Currently, ADR is one of the most robust colonoscopy quality indicators, and is notably associated with a lower risk of CRC between two colonoscopies [15]. In a French study on 2,000 colonoscopies, Coriat et al. found an ADR of 31%, although this number only represented complete colonoscopies [7]. In the setting of CRC screening, Denis et al. found an ADR of 30% [12] in accordance with the objectives proposed by Blanks et al. [16]. However, larger studies reported ADRs closer to 20%, as in the present study [9,17]. This relatively low ADR might be explained by several factors: poor bowel preparation, as suggested by the cecal intubation rate <90%; and the variety of indications for the colonoscopies, with a quarter of the patients either younger than 50 or having a colonoscopy for abdominal pain or changes in bowel habits. A recent study on more than 12,000 colonoscopies revealed that the patients’ gender and age, the quality of bowel preparation, the level of endoscopists’ continuing medical education, and the quality of the endoscopic devices, were factors associated with the ADR [18]. Here, the patients’ gender and age (over 50), personal or family history of intestinal lesion, and FOBT positivity were significantly associated with a higher ADR. These data support the current French CRC screening program, in which colonoscopy is performed in patients with either positive FOBT or personal or familial history of colorectal cancer or adenomas, i.e. in patients in which the ADR will be the highest. The type of bowel preparation also influenced the ADR, with a significant variation from 14.8% for sodium picosulfate-based preparations to 20.2% for PEG-based preparations (p=0.002). It is unclear why a bowel preparation with sodium picosulfate was associated with lower ADR values in the present study. A reduced laxative effect or insufficient dosage of sodium picosulfate may be implicated. More recent use of these protocols, leading to insufficient explanations to patients from endoscopists, or suboptimal compliance with the 1-1.5L additional fluid intake requested by this protocol could also explain the results. However, the choice of the bowel preparation was not justified by the participating gastroenterologists and possibly in relation with prescription habits. Furthermore, the quality of bowel cleansing was not assessed by specific scores. Therefore, the difference observed in ADRs among the various bowel preparation regimens may be explained by the age of the patients or the indication of the colonoscopy rather than by the quality of the bowel preparation. In conclusion, the PEG-based split-dose bowel preparation seems to remain an acceptable standard, as confirmed by other recent work [19].

French law currently forbids gastroenterologists (and non-anesthesiologists) from administering anesthetic drugs such as propofol. This explains the very high proportion of colonoscopies performed under general anesthesia and monitored either directly by an anesthesiologist, or with the help of a specialized nurse (90.2% of all procedures). These results contrast with the numbers reported by North American [20] and other European authors [21–23]. However, considering the increasing number of colonoscopies performed worldwide, anesthesiologist capacity is very likely to become insufficient. Therefore, the proportion of sedation with propofol administered by a non-anesthesiologist may increase in the future.

The number of high-grade adenomas or adenocarcinomas increased with the size of the polyp and reached 24% for lesions of 10 mm and larger, in accordance with previous reports [24]. However, almost 2% of polyps of 5 mm or less (usually referred to as diminutive polyps) presented worrying histological features. This number is notably higher than the proportion of 0.5% reported by Gupta et al. in a large retrospective analysis [25]. Among non-invasive screening tools for CRC, colon capsule endoscopy shows a sensitivity of 60% [26], and CT colonography has been proven to be unreliable for the identification of such polyps [27], as well as flat ones [4]. This point should be emphasized, because some authors propose that such polyps should be resected and discarded [28]. Conversely, our data suggest that an *in vivo* characterization of diminutive polyps should be performed in order to obtain a histological analysis of each resected lesion, regardless of its size, and to propose a follow-up of benign, diminutive polyps.

Finally, the higher polyp detection rate noted in patients with a previous colonoscopy can be viewed in relation to the prevalence of CRC in our patients. Indeed, our results support the hypothesis that colonoscopy-based CRC screening programs in patients with a personal history of colorectal polyps lead to repeated polyp resections, thereby protecting patients from the onset of CRC, since we observed a three-fold reduction in CRC prevalence in patients with previous colonoscopy (4.2% *vs.* 1.4%; p<0.0001).

Our study has some limitations. First, it relies on the hypothesis that the colonoscopies performed during the selected week were representative of the weekly endoscopic activity of French gastroenterologists. Second, in spite of a rigorous online questionnaire in which all items had to be recorded before validation, there may be some bias due to the selection or loss of information among participating physicians. The proportion of unavailable histological results would support this point (10.8%). Furthermore, the completeness of the reports could not be assessed. Third, a potential sampling bias among the population of endoscopists should also be mentioned, because of the low participation rate of 13%. This could explain the overrepresentation of private practitioners in our study. However, 2011 data from the French national health insurance fund indicate that about 70% of colonoscopies are performed by private practitioners in France [14]. Fourth, recording data for only one week is useful in optimizing physicians’ adherence to the survey, but this time period may be too short to record rare events such as colonic perforations occurring outside the context of therapeutic colonoscopy. This complication usually occurs in 1/1,000 of colonoscopies, but was not reported even once here.

CRC screening in France in the average-risk population relies on FOBT (Hemoccult^®^) every two years, and complete colonoscopy is limited to either positive FOBTs or populations at a high risk of CRC. Nevertheless, participation in CRC screening programs based on FOBT results remains low (32% in 2011 in France [29]). The higher ADRs found in the CRC screening subgroup highlight the importance of colonoscopy as a screening tool for CRC. Endoscopy might even play a role as a CRC screening tool as first-line strategy, as is the case in numerous other countries [30]. Our results show that colonoscopy in 2011 in France is a safe procedure with a high diagnostic yield, especially in the setting of CRC screening. In accordance with very recent studies [3,31] our data suggest that colonoscopy and colorectal polyp resection could contribute to a significant reduction in the incidence of CRC. The use of anticoagulant or antiplatelet drugs appears to be safe, as suggested by the absence of hemorrhagic complications in patients treated with these drugs. In addition, the rate of colonic perforations noted (0.1%), reveals the progress accomplished in the practice of endoscopy (perforations were restricted to therapeutic colonoscopies). However, improvements remain to be achieved in identifying some of the major quality criteria for colonoscopy, such as bowel preparation and cecal intubation rate. Further studies with a larger number of gastroenterologists will enable clinicians to monitor these improvements. Our study confirms the paramount importance of colonoscopy in the prevention of CRC, with one out of five colonoscopies in France leading to the diagnosis and resection of a colorectal adenoma.
